# Globalized Religious Aftershock at the Beginning of the Twentieth Century—the Apapocúava-Guaraní Cataclysm and the 1906 San Francisco Earthquake

**DOI:** 10.1007/s41603-023-00189-7

**Published:** 2023-01-23

**Authors:** Stefan van der Hoek

**Affiliations:** Faculty of Theology, Friedrich-Schiller University Jena, Fürstengraben 6, 07743 Jena, Germany

**Keywords:** Nimuendajú, Earthquake, Media, Globalization, Modernity, Religion

## Abstract

The impact of environmental catastrophes and crises on religion and religious discourses in history and modernity has been described frequently and from different perspectives. However, the interpretations and narratives of scriptless civilizations have remained largely unnoticed. Due to the concrete lack of reliable sources of information, those interpretations and narratives can nowadays only be recorded and processed in the scientific discourse in a fragmentary way. Therefore, this article unfolds along the early work of ethnologist and linguist Curt Unckel (1883–1946), who was called Nimuendajú during his lifetime, the thesis that an indigenous group of the Apapocúava-Guaraní tribe in southeastern Brazil correlated the global information dissemination of the 1906 San Francisco earthquake with cosmological narratives of an impending apocalypse, leading to the decline and cultural degeneration of the group. The article thus demonstrates how cosmologies and world perceptions of an indigenous tribe at the dawn of globalization can be reconstructed and how information about catastrophic events from the news was processed with immediate local changes by a scriptless culture on the Brazilian frontier in the early twentieth century. In doing so, this article examines the role of media and communication in globalized modernity and how media literacy influences religious perception.

## Introduction

Environmental catastrophes and calamities such as tsunamis and earthquakes have repeatedly raised the question of the metaphysical or supernatural. The meaningfulness of crises is being asked anew in human history. Accounts of such catastrophes and their respective interpretations are preserved especially in religious discourses. In some cases, the authors of such texts and traditions provided an apocalyptic undertone concluding that the events are a punishment or a warning from the God(s) or/and that “the end of the world is near.” In other cases, worldviews and cosmologies arrive at different interpretations in their narrative traditions, which rather help to deal with unknown events and provide coping strategies. Such strategies can be related to the notion of the functionality of religion. In this understanding, religion serves to deal with crises and other unexpected events, as could be observed in many cases worldwide during the COVID-19 crisis (van der Hoek 2022a). Religion can from a functional perspective also serve to strengthen individuals psychologically in crises and provide resilience (Herzog et al. 2020: 15) or help to deal with different world views (Nagel 2021: 221). For groups and larger societies, religion functions as a resource to cope with contingency and create shared values (Dutra and de Almeida Sales 2018: 23). Therefore, it is not uncommon for crises such as environmental catastrophes to be placed in a religious causal context and for answers to be sought in religions (O'Mathúna 2018: 29). However, some scholars would argue that religious interpretations would have played a role in particular for ancient societies who attributed the crisis to acts of gods but could be nowadays better be understood as a complex nexus of natural–human–social–economic factors (Chaudhary and Piracha 2021: 1124). Those who think that religious explanations for natural catastrophes are exclusively related to ancient worldviews are far wrong, however. In contemporary discourses, the link between environmental disasters and religion is proving to be a surprisingly long-lasting alloy. In particular, the global dissemination of information through media has led to new dynamics and an enormous increase in religious discourses related to environmental disasters. This was e.g. evident in the case of the Haiti earthquake in 2010. The earthquake was a devastating catastrophe that occurred at 21:53 UTC on January 12, 2010. Its hypocenter was about 15 miles west-southwest of the Haitian capital of Port-au-Prince at a depth of about 8 miles, it lasted only 35 s, and took the lives of about 84 people. US Pentecostal Pat Robertson, who was not personally affected by the destructive earthquake but learned about it from the global news, immediately blamed the population of Haiti for it. Robertson interpreted the dramatic events as God’s punishment for a pact with the devil that the people of Haiti allegedly concluded (Anderson 2013: 208). Representatives from other religious communities across the world, such as Judaism, Buddhism, and Islam, also linked the 2010 earthquake in Haiti as well as the 2004 tsunami in Thailand to the sins of the people in their respective places (O'Mathúna 2018: 28). Also, among scholars’ connections between God and catastrophes can be found. Anthropologist David C. Lewis presented recently a thesis linking the 1988 earthquake in Armenia to biblical prophecies from the Book of Amos. Lewis wondered what God wants to tell people through earthquakes. In doing so, Lewis points to the social and political parallels between the ancient Israelites and contemporary Armenia, projecting the divine message of conversion and repentance to the Armenian population into the modern era (Lewis 2022: 28).

For describing Latin American religions, the question of how earthquakes and environmental disasters are interpreted can mean different approaches. A separate thematic issue on religion and pandemics for example was recently published in the *International Journal of Latin American Religions*. In its introduction, the editors stated that global media coverage is an important role in the perception of environmental disasters (Usarski and Py 2020: 165). In this article, I will present that the role of global knowledge transfers thru media coverage about earthquakes can be detected as early as the beginning of the twentieth century even among indigenous groups living in the interior of São Paulo. From a historical perspective, this period can be described not only as the transition to industrialization and urbanization but can also be understood as a starting point of digital literacy (Ayhan 2017: 33). As Hartmut Rosa pointed out, the elements of globalization in modern times cannot only be described by its long-distanced transfer of ideas, goods, and people, but rather by its velocity and the reduced resistance in the process of transfer (Rosa 2013: 214). The invention of the telegraph in the nineteenth century made it possible to send information around the world in a remarkably short time. The spread of information about wars, epidemics, and natural disasters also spurred the interpretation at this time that God had a message to mankind and wanted to put a stop to man’s drive for more power through machines, technology, and science. Similar to the Tower of Babel (Genesis 11), God descends from heaven and causes confusion and destruction among civilizations that presume to act as God themselves, at least in a Christian or Jewish interpretation of contemporary events. Religious discourses can be manifold in this context as accompaniments of modernity and industrialization for various examples such as the Titanic, the Spanish Flu, or the First World War, just to name a few.

The particular focus of this article is the 1906 San Francisco earthquake and the question of how an indigenous tribe of the Apapocúava-Guaraní in the state of São Paulo interpreted the news of the earthquake. For this purpose, I analyze an article written by the anthropologist and linguist Curt Nimuendajú Unckel, who conducted his research data among different indigenous tribes and published them in a German-speaking journal of ethnology (*Zeitschrift für Ethnologie* which is today formally known as *Journal of Social and Cultural Anthropology*) in 1914. Nimuendajú’s article deals with the sagas of the creation and destruction of the world as a basis of the Apapocúava-Guaraní religion and provides detailed information about the worldview of an indigenous group in the state of São Paulo at the beginning of the twentieth century and how they incorporated information from newspapers into their world view. The time in which Nimuendajú lived among the Apapocúava-Guaraní and collected the research data falls into the period in which many globalization theorists locate the beginning of globalization (Robertson 1990: 17). Peter Worsley had already pointed out that the idea of a global human society first emerged during the period of European colonialism (Worsley 1977: 14). Media played a central role in the emergence of consciousness of a world society into a singular social system, since media is a relevant vector for global communication and must therefore be understood as the basis of world society (Luhmann 1982: 133). Media and globalization seem therefore to reinforce certain dynamics in the religious field that will be examined in more detail.

## Nimuendajú and the Research on the Apapocúava-Guaraní

Curt Nimuendajú Unckel began his fieldwork among the Apapocúava-Guaraní in 1905. At this time, he had been living in Brazil for just 1 year. Originally, he was born on April 17, 1883, as Curt Unckel in Jena, which is a comparatively small town in what is now the federal state of Thuringia. Unckel grew up in Jena and served an apprenticeship at Carl Zeiss Factory. Following his apprenticeship, which he finished successfully in 1903, Unckel emigrated to Brazil. When Unckel arrived in São Paulo, he was 20 years old and initially worked as a salesman in a hardware store in Santa Ifigênia in São Paulo (Dungs 1991: 18). The store was located on Florencio de Abreu Street and belonged to Ricardo Naschold, who was also of German origin. The store was located near the German school and a Lutheran church, which was particularly frequented by German emigrants.

At the beginning of 1904, Unckel undertook his first expeditionary voyages and joined an adventurous group that set out on an exploratory expedition across the Rio Feio that lasted several months in order to attempt to free Catholic missionaries from the captivity of an indigenous tribe of the Coroado. No exact details are known about the expedition, but it represented a key moment in Unckel’s fascination with the study of indigenous peoples and the future of his life (Dungs 1991: 19). Unckel returned to São Paulo with a pierced lower lip that was interpreted as a sign of belonging to an indigenous tribe (Capeller 1962: 17). From then on, Unckel made regular trips to various indigenous tribes, such Caingangues, Ofaié, and Terena groups and remained with the Apapocúava-Guaraní in the state of São Paulo until 1907.

During his stay among the indigenous group, Unckel became adopted by a family of the Apapocúava-Guaraní and received thereby the name “Nimuendajú.” The name can be translated as “who makes his own home,” “who made his home among us,” or “who came to sit down among us.” It is not clear when Unckel used this name for his correspondence and identified himself with it. From 1912 on, however, Unckel signed his letters and official documents as Nimuendajú. Curt Unckel’s naming and name change to Nimuendajú marks a dominant feature of his research approach, which is described by Schröder as follows: “In terms of historical comparison, Nimuendajú privileged to some extent ‘the native point of view’ even before Malinowski announced this noble objective for anthropology” (Schröder 2019: 346). Among the Apapocúava-Guaraní, Nimuendajú became more intensely acquainted with the indigenous way of life and began his documented fieldwork. Until 1913, Nimuendajú undertook various expeditions to different indigenous groups in the states of São Paulo and Mato Grosso. He served also the *Serviço de Proteção aos Índios* (SPI) and conducted research for different museums and institutions. In 1913, Nimuendajú emigrated from São Paulo to Belém in the state of Pará. In all likelihood, the move to Belém was made at the suggestion of the director of the SPI, Marshal Cândido Mariano da Silva Rondon (1865-1958). In Belém, Nimuendajú had his permanent residence until the end of his life (Schröder 2019: 345). Nimuendajú undertook numerous expeditions, working as an employee of the SPI and occasionally as a freelancer for the *Museu Paraense* (Dungs 1991: 24–25). Through the mediation of the director of the *Museu Paraense* (now *Museu Paraense Emilio Goeldi*, MPEG), the ornithologist Emilie Snethlage (1868-1929), contacts were made with German academic ethnology, which opened the doors to the publication of Nimuendajú’s first scientific article on the religion of the Apapocúava-Guaraní (Schröder 2019: 346). In this article, Nimuendajú reveals insides about the curious events that happened after the indigenous group received the news about a devastating earthquake.

## The Settlement Area of the Apapocúava-Guaraní at the Beginning of the Twentieth Century

The region where Bauru is located and near which the indigenous Apapocúava-Guaraní tribe lived during the period when Nimuendajú conducted his research, was historically a contested area between the Caingangues and the Guaranis. It was not until the eighteenth century that the Brazilian Bandeirantes arrived to settle in the region (Franco 2018: 187). The region was favorably read geographically for further expansion, as it marks a crossing point for rivers heading to the states of Mato Grosso and Goiás. However, the navigation of the rivers was hindered by constant attacks from the local indigenous tribes. Therefore, the Bandeirantes did not manage to settle in the region until the nineteenth century. The establishment of permanent habitation projects by the Brazilian pioneers became only possible due to the intensified settlement policy, through which settlers from the regions of the state of Minas Gerais and Rio de Janeiro were targeted to settle in the interior of São Paulo. After 1850, other pioneers from São Paulo and Minas Gerais, in search of new land for colonization, began to explore the vast regions between the Botucatu mountain range, the Tietê River, the Paranapanema River, and the Paraná River, which until then had been inhabited almost exclusively by indigenous groups. In 1856, Felicíssimo Antônio Pereira and Antônio Teixeira do Espírito Santo acquired lands in the area and founded the Fazenda called Flores near the present-day center of Bauru. In 1884, part of this Fazenda, also called Campos Novos de Bauru, was transformed into the settlement of São Sebastião do Bauru. The district continued to develop despite its relative isolation from the rest of the state and the attacks of the Caingangues. In 1888, Bauru become a district of the city of Agudos and in the following year, the railroad tracks of the Sorocabana-Botucatu line reached the region and caused a price explosion of land valuations and attracting more immigrants from the coastal regions (Ghirardello 2002: 79). The arrival of more immigrants from São Paulo and Minas Gerais led to the emancipation of the municipality of Bauru on August 1, 1896 (Ghirardello 2002: 70). During the time in which Nimuendajú lived among the Apapocúava-Guaraní, this region of São Paulo state was undergoing massive deforestation. In Bauru and other municipalities, immigrants were encroaching on the forests and destroying the livelihood of indigenous peoples. In addition, at the beginning of the twentieth century, Brazil saw a sharp increase in agricultural production. This had to do with the fact that Brazil sought to satisfy the increased demand for agricultural products on the world market to stabilize and increase its economy (Cano 1988: 6).

At that time, Bauru had a population of just 3000 people, and over the course of the twentieth century, it developed into a city with over 300,000 inhabitants in a remarkably short period (Pinheiro and Danni-Oliveira 2012: 49). More than in any other state in Brazil, industrial agricultural production increased in São Paulo during this period and expanded inland (Cano 1988: 11). Bauru developed into one of the most productive regions for coffee production in the state of São Paulo by the 1950s (Cano 1988: 46), which had the effect of depriving the indigenous tribes in the region of their livelihood.

## The San Francisco Earthquake and its Religious Interpretations

In the definition of this research article, I will now turn to the events that took place in San Francisco in 1906 and relate them to the global media coverage that reached the indigenous tribe to provide a link to Nimuendajú’s findings mentioned above.

The San Francisco earthquake shook the coast of Northern California on April 18, 1906, and is considered one of the worst natural disasters in the history of the USA. More than 3000 people died in San Francisco and its surrounding area. The epicenter of the main quake was located less than two miles away from the city center, near Mussel Rock. The quake shook the area along the San Andreas Fault and was felt from Oregon to Los Angeles and as far away as Nevada. Just as destructive as the earthquake itself were the resulting fires that broke out in many neighborhoods. Most of them resulted from destroyed gas lines, furnaces, and fireplaces, but some were the result of arson. Since many insurance policies only covered fire damage but not earthquake damage, residents would set fire to their own dwellings in order to get the damage compensated by the insurance. In addition, the blasting of buildings was used explicitly by the government to prevent fires from spreading through the densely populated city. However, because of ignorance, incompetence, and the lack of suitable explosives, in many cases even more fires were started.

Similar to the earthquake of Lisbon in 1755 and the earthquake of Caracas in 1812, various religious discourses entwine around the events, placing the natural disaster in the context of eschatological prophecies or wondering what God wants to say to the people (McCook 2009: 53, Sandin 2018: 15). Especially the catastrophe of the Lisbon earthquake and destruction was followed by a tremendous “religious aftershock”, which Lutzer describes as follows: “Just as earthquakes create aftershocks, natural disasters create religious aftershocks. Believers wrestle with doubts; unbelievers use disasters as justification for their refusal to believe in a loving God” (Lutzer 2011: 5).

Religious aftershocks can also be identified around the San Francisco earthquake, but in contrast to the 1755 and 1812 earthquake, they have spread further and circulated the globe within a very short time due to the worldwide use of media agencies and new technologies such as the telegraph. Therefore, the news of the earthquake in San Francisco were published the very next day in the local press in Bauru.

The San Francisco earthquake has fueled religious interpretations behind the horrific event worldwide. Arguably the most prominent and far-reaching narrative within religious studies, the early development of the *Azusa Street Revival* can be seen as a religious interpretation related to the San Francisco earthquake, although its significance to the global Pentecostal movement must be doubted (Davis 1999: 97). Early in 1906, African American preacher William J. Seymour (1870-1922) immigrated to Los Angeles to preach the message of the second baptism by the Holy Spirit. In a small barn on Azusa Street in Los Angeles, believers from different countries of origin, races, and social classes gathered and celebrated ecstatic and noisy worship services in an egalitarian community (Anderson 2013: 45). Local journalists from the US West Coast covered the curious events on Azusa Street in the *Los Angeles Daily Time*, mocking the practices of the parishioners and especially the peculiar missionary Seymour in the article titled “*Weird Babel of Tongues*” (Kay 2011: 25). The very next day, as the malicious article was printed in the newspaper daring to mock the church gathering on Azusa Street, the city of San Francisco was destroyed by the massive earthquake (Liardon 2006: 98). Many adherents inside the young US-american Pentecostal movement interpreted this as a direct response from God to the critical media coverage a day earlier (Kay 2011: 25).

While religious interpretations of the events were frequently covered by the Christian-based public in the context of the Azusa Street Revival movement, significant religious interpretations also took place in San Francisco’s Chinatown among the Chinese diaspora. Two repeated interpretations involve, first, the earth dragon *Day Loong*, which San Francisco’s Chinese immigrants supposedly believed to be behind the quake and has caused devastating damage to the city. Another interpretation among the Chinese immigrants had to do with a herd of cattle that, startled by the disaster, ran through the streets of San Francisco. Although all the cattle were captured, one of the bulls got lost in the streets of Chinatown, and the appearance of the animal amid the crisis aggravated the situation because it was taken by the Chinese as a negative omen. The Chinese immigrants suspected that the bovine might be one of the four bulls on whose back, according to a Chinese myth, the earth rested. The earthquake was thus associated with the cow that had lost its way and therefore caused the earthquake (Birken 2018: 26).

## The Apapocúava-Guaraní and the Interpretations of Catastrophes in the Horizon of Cataclysm

In the summer when Europe entered the First World War, an article by an author with the foreign-sounding name of Nimuendajú was published in the German-language academic journal “*Zeitschrift für Ethnologie*.” The title of the 121-page article was “*The legends of the creation and destruction of the world as foundations of the Apapocúva-Guaraní religion*.” Even though Nimuendajú had written an extensive monograph based on his deep and long-standing experiences among the Apapocúava-Guarani, he was a self-educated researcher without any formal academic education (van der Hoek 2022b: 108). This was Nimuendajú’s first academic publication and included empirical data basis participant observations and recorded narratives, which Nimuendajú analyzed according to his method, which he described in detail. Accordingly, the data were conducted through the narratives of different individuals within the group. In order not to unconsciously falsify the narratives and to make the research results intersubjectively comprehensible, Nimuendajú repeatedly listened to the verbatim dictated narratives and had them retold by different persons again and again. Nimuendajú reflects and justifies in detail that his interpretations required intersubjective confirmation, writing:

„Ich habe stets wie ein Indianer unter Indianern gelebt und dabei, wenn auch nicht durchaus fehlerfrei, so doch vielleicht besser Guarani sprechen gelernt als mancher, der darüber mehr geschrieben hat als ich. Die Mythen, welche ich hier behandeln werde, habe ich unzählige Male teilweise, seltener ganz, nicht nur erzählen gehört, sondern auch selbst erzählt.“ (Nimuendajú 1914: 284)

“I have always lived like an Indian among Indians and have learned to speak Guarani, if not without mistakes, perhaps better than many who have written more about it than I have. The myths, which I will treat here, I have countless times not only heard partially, rarely completely, told but also told myself.” (Translation: SvdH)

Subsequently, when the narratives were recorded and documented by different speakers, the narratives were logged and no longer revised so as not to falsify their content retrospectively. In his work, Nimuendajú was assisted by three members of the group, with whom he formed a research team. In his descriptions of the composition of the research group, Nimuendajú attaches great importance to showing the different character traits of each member in a well-founded manner and making his role within the group transparent.

„Meine Gewährsleute waren drei gute Freunde aus der Apapocúava-Horde, der ich selbst ja auch angehöre: der alte, konservative Guyrapaiju, der vielgereiste Tupäjü und besonders der religiös-schwärmerische Joguyrov.“ (Nimuendajú 1914: 285)

“My confidants were three good friends from the Apapocúava horde, to which I belong: the old, conservative Guyrapaiju, the well-traveled Tupäjü, and especially the religiously enthusiastic Joguyrov.” (Translation: SvdH)

Nimuendajú focused his research question primarily on religion and its practice under the conditions that the group was experiencing at the time. To obtain research data and to create an adequate sample, Nimuendajú primarily used the experts of religion, whom he preferred to interview and accompany during the practice of religious and cultural acts:

„Ich habe mit Vorliebe die Gesellschaft der Alten und besonders der Medizinmänner gesucht und mich mit ihnen viele Stunden lang über die alte Religion belehren lassen.“ (Nimuendajú 1914: 285)

“I have preferred the company of the ancients, and especially of the medicine men, and have spent many hours with them being instructed in the old religion.” (Translation: SvdH)

Nimuendajú starts his argument with the observation that the traditional religions and world interpretations of the Apapocúava-Guaraní had been preserved astonishingly despite the Jesuits’ missionary attempts over many years and continued to present themselves as living phenomena in their communities (Nimuendajú 1914: 284). Furthermore, he reports, that at the beginning of the nineteenth century a religious movement emerged that was initiated by charismatic leaders and lasted until the author’s present time:

„Durch Visionen und Träume inspirierte Medizinmänner traten als Propheten des nahen Weltuntergangs auf, sammelten in mehr oder weniger großer Zahl Anhänger um sich und begaben sich mit diesen unter Medizintänzen und Zaubergesängen auf die Suche nach dem „*Land ohne Schlechtes*“. Welches einige, der Tradition zufolge im Mittelpunkt der Erde, die meisten jedoch im Osten, jenseits des Meeres vermuteten. Nur so hofften sie dem drohenden Verderben zu entrinnen.“ (Nimuendajú 1914: 287)

“Inspired by visions and dreams, medicine men appeared as prophets of the near end of the world, gathered followers around them in more or less large numbers, and went with them under medicine dances and magic chants on the search for the ‘*land without evil*’. According to tradition, some believed it to be in the center of the earth, but most believed it to be in the east, beyond the sea. Only in this way, they hope to escape the impending doom.” (Translation: SvdH)

The *land without evil* is a frequently used motif that can also be found among indigenous Guaraní groups in northern Brazil (Wright 2017: 46). By the research of Nimuendajú about the religion of the Apapocúava-Guaraní, the *land without evil* is described as a paradisiacal place where one could not die nor suffer from any pain (Nimuendajú 1914: 287). As a result of these prophecies about the *land without evil*, a messianic migration movement began among the indigenous population, which had different phases, directions, and starting points. However, the Brazilian state was dissatisfied with the incipient migratory movements of the indigenous population and was suspicious of them. Concrete measures were therefore initiated by the government to push the indigenous tribes back into their original settlement areas and nip further migratory movements in the bud (Nimuendajú 1914: 288–289). In addition to the state pressures, plagues and epidemics further decimated tribal populations, resulting in the infection of germs and viruses brought by European immigrants through increased contact. The decimations meant that by the time of Nimuendajú’s research visits, there were only a few about 100 members of the Apapocúava-Guaraní left that maintained inside the community (Nimuendajú 1914: 290). The few members of the group spent their time on reservations and were threatened in their existence by diseases and invasions by other tribes (Nimuendajú 1914: 292–293). Shortly before Nimuendajú’s arrival, a final attempt was made in 1905 to find the *land without evil*, but this expedition also had to be unsuccessfully abandoned after a short time due to adversity (Nimuendajú 1914: 293). When Nimuendajú came across, the group was disillusioned about finding the *land without evil*.

After this description of the situation, a large part of Nimuendajú’s research deals with the religion, mythologies, and contemporary interpretation of the Apapocúava-Guaraní. Of the 121 total pages in the article, 88 pages alone are devoted to the chapter on the Apapocúava-Guaraní and their religion and cosmology. This illustrates the great attention that Nimuendajú devotes to the subject of religion in his descriptions.

Nimuendajú begins by describing the use of Christian symbols such as crosses, sacred figures, baptismal fonts, and Christian language, which the Apapocúava-Guaraní had appropriated in recent centuries and used primarily as protective mechanisms against attacks and killings by the Brazilians, who recognized only those as “real” human beings who became Christian (Nimuendajú 1914: 299; 379). For the Apapocúava-Guaraní themselves, these symbols were primarily superficial in character. Rather, they serve to identify themselves as Christian to missionaries to let the uninvited guests leave as quickly as possible without causing a stir (Nimuendajú 1914: 300). This shows, moreover, that Nimuendajú was trusted by the Apapocúava-Guaraní, who gave him a place in their religious activities, they revealed secret myths to him, and allowed him to observe them with participation. From his observational role, Nimuendajú vividly describes in detail how dreams influence the knowledge and decisions of the Apapocúava-Guaraní (Nimuendajú 1914: 306–307), how the individual components of the soul affect the native’s interpretation of the world, and what happens to the individual components of the human being after death (Nimuendajú 1914: 308–309). Nimuendajú’s analysis goes even beyond a mere repetition of what is observable; rather, he places the narratives in context. He spans a wide range of tensions in which the actions and narratives are embedded in religious semantics. For example, Nimuendajú describes the Apapocúava-Guaraní creation myths (Nimuendajú 1914: 316–318), which describe various creatures such as the blue jaguar, giant mosquitoes, and other venomous animals. These creatures often arise from the stories themselves and are not in all cases attributable to a God who has created them (Nimuendajú 1914: 327). Nimuendajú discusses Apapocúava animism at length (Nimuendajú 1914: 301–316) and addresses the deification of individual healers (Nimuendajú 1914: 327–329).

The narrative that is particularly relevant to this article is embedded in the context of the annihilation of the world about its creation. In Apapocúava-Guaraní mythology, the world was created by Nanderuvuçú. In the beginning, he set up a foundation for the earth by crossing one beam in a direction from east to west and another in a north–south direction. Nanderuvuçú filled the empty spaces between the crossed wooden beams with earth, upon which forests grew, rivers, oceans, and lakes were formed and human beings settled. The destruction of the world would be initiated by Nanderuvuçú’s brother, called Nanderzqueý. Nanderzqueý is a similar powerful God who will appear at the end of time and his destiny would be to slowly pull out the eastern end of the crossed wooden beams. This will cause the earth in the west to lose its hold and gradually fall into an abyss. Therefore, the earth will begin to burn from its lower side in the west, and fire will spread over the earth (Nimuendajú 1914: 332). This will initiate the destruction of the world, which will continue with a flood and the appearance of the blue jaguar. The blue jaguar is a powerful deity that will descend from heaven in order to destroy all human beings (Nimuendajú 1914: 333–334).

Nimuendajú describes how the Apapocúava-Guaraní completely abandoned their hopes for the realization of their arrival to the messianic *land without evil* after they received news from a member of their tribe in 1906. One young man of the group delivered the message from Bauru that it was written in a newspaper that a part of the world had crashed and gone up in flames. The Apapocúava-Guaraní had no doubts that this message from the newspaper was related to their own myths and that the end of the world would come now (Nimuendajú 1914: 335). Accordingly, it was only a matter of time until the earth would collapse, the fire would come upon them, or the blue jaguar would finally destroy them. Nimuendajú pays no further attention to the event of the transmission of the message from Bauru itself and focuses on the immediate impact of the news regarding its religious interpretations. From a descriptive position, as the situation on the ground presents itself in the article, it is of particular interest in retrospect to ask about the more specific events that this message may have triggered in 1906.

Archival newspaper articles indicate that coverage was also reported in Brazil in connection with the San Francisco earthquake on April 18, 1906. The newspaper *Correio Paulista*, which was widely distributed and read in the state of São Paulo at this time, reported on April 19, 1906, under the headline “*Tremenda catástrofe*” (English: *tremendous catastrophe*):

“Despachos agora recebidos de Washington e de S. Francisco da California dizem que esta cidade acaba de ser quasi totalmente destruida por pavoroso terremoto. Sabe-se que ha centenas de mortos, attingindo a milhares os feridos. Os prejuizos são calculados em cerca de trinta milhões de dollars. Outra parte da cidade está ameaçada de ser devorada por medonho inçendio. Os bombeiros e populares trabalham desesperadamente. Novos despachos chegados á tarde de S. Francisco da California asseguram que a população daquella cidade está fugindo transido de pavor, receiando a replicação do terremoto. É horrível o espectacolo que apresenta a cidade que se acha a meio em escombros e presa das chammas.”^1^

“It has just been reported from Washington and San Francisco, California, that this city has been almost destroyed by a terrible earthquake. It is known that hundreds of people were killed and thousands injured. The damage is estimated at thirty million dollars. Another part of the city is in danger of being engulfed by a terrible fire. Firefighters and people are desperately working. New reports reaching us this afternoon from San Francisco, California, assure us that the population of that city is fleeing in fear of a repeat of the earthquake. It is a terrible spectacle that the city, half in ruins and trapped by flames, presents.” (Translation: SvdH)

The information that a part of the world in the west had collapsed and gone up in flames may make the tribe conclude that the end of the world is near. As a consequence, the tribe gave up hope for the fulfillment of the prophecy that there would be a *land without evil*, which could provide a safe haven for them. They surrendered to their fate and fell into a cultural pessimism that embedded itself with the looming decline of the way of life and threats from the outside due to growing migration and alienation.

This event is of particular interest to the argument of this article, showing how globalization and mediatization can inspire religious imaginaries and influence religious interpretations even in indigenous tribes on the periphery of so-called civilization. Thus, such a correlation points to the fact that the influence of mediatized communication at the early time of globalization could also play a significant role for indigenous cultures on the periphery, in that they viewed global accounts through the lens of their cosmologies.

Paradoxically, the Apapocúava-Guaraní tribe tried to deny themselves outside influence by accepting and externalizing Christian symbols and language. But it was precisely through the global dissemination of media that the cosmology of the worldview was shaken and the prophecy of the approaching end of the world was expected.

In Nimuendajú’s further visits to the group in 1912, the stories of the near end of the world associated with the reporting of 6 years earlier were still virulent, and the members of the group expected the end of the world in the very near future (Nimuendajú 1914: 335).

## Conclusion

To conclude this article, it remains to reflect on the fact that there can be no conclusive confirmation that the indigenous Apapocúava-Guaraní tribe saw the end of the world approaching through the news of the San Francisco earthquake and whether this event led to the extinction of the tribe. After 1912, the trace of the tribe is lost and cannot be reconstructed further. Central to this is the religion-specific moment that the messianism of the Apapocúava-Guaraní was not directed toward a God who would bring salvation. Rather, it was up to the Apapocúava-Guaraní themselves to find the *land without evil*, which they failed to accomplish.

Just as Wright already stated about the indigenous tribes in northern Brazil, similar could be said about the tribes in the State of São Paulo: “[…] indigenous societies have suffered transformation, often catastrophic, since the beginning of the colonial regime (epidemics, demographic collapse, atrocities) – so severe that it is hardly imaginable that these have taken place without leaving deep and structural traces within their cosmologies and socioreligious processes as we have come to know them” (Wright 2017: 44).

However, it must be noted that the indigenous tribes in the region might still have managed to resist the invasion of the Bandeirantes and to mutually constitute themselves as a community or find a place to survive in another region. What led to the tribe’s extinction, however, was not a disease and physical annihilation, but the tribe’s religious worldview that did not provide hope for a better future somewhere else. Confronted with the globalized circulation of news and information, the tribe expected the immediate annihilation of the world. To return to Luhmann’s theory of globalization, the contact between the indigenous tribes and Western civilization boiled down to the fact that only one of the social systems could survive and that communication across the globe plays a crucial role in this regard: “Under modern conditions, however, and as a consequence of functional differentiation, only one societal system can exist. Its communicative network spreads over the globe. It includes all human (i.e., meaningful) communication […]. A plurality of possible worlds becomes inconceivable” (Luhmann 1982: 132–133).

The passive defeat and lack of search for real or spiritual liberation among the Apapocúava-Guaraní can only be understood as a side effect of globalization in this context. Forced to keep up with the accelerated global spread of information, the Apapocúava-Guaraní could not adapt their religious interpretations to the velocity of the modern world. While religious interpretations about the end of the world in the Pentecostal movement and the Chinese diaspora in San Francisco faded over time, the Apapocúava-Guaraní hold on to the conviction that the end of the world is near. The different velocities of the two civilizations may have led to the dramatic extinction of the slower one when they came into contact. Thus, it can be shown that religion plays a fundamental resource in the global processing of media and that the increased speed with which news circulates the world requires from members of modern societies capacities which are not a priori inherent in human beings but are appropriated, learned, and incorporated in the course of socialization within modernity.

## Data Availability

Not applicable.

## Footnotes

1 Correio Paulista, April 19, 1906. Available at: < http://memoria.bn.br/pdf/090972/per090972_1906_15327.pdf > Access 2 Nov. 2022.
